# Hypertension and its related factors among patients with type 2 diabetes mellitus – a multi-hospital study in Bangladesh

**DOI:** 10.1186/s12889-022-12509-1

**Published:** 2022-01-29

**Authors:** Hiba Alsaadon, Afsana Afroz, Afsana Karim, Samira Humaira Habib, Mohammed J. Alramadan, Baki Billah, Aishwarya Narendra Shetty

**Affiliations:** 1 Department of Epidemiology and Preventive Medicine, School of Public Health and Preventive Medicine, Faculty of Medicine, Nursing and Health Sciences, Monash University, 553 St. Kilda Rd., Level 4, Melbourne, VIC 3004 Australia; 2 Bangladesh Institute of Research and Rehabilitation in Diabetes, Endocrine and Metabolic Disorders (BIRDEM), Dhaka, Bangladesh

**Keywords:** Bangladesh, Type 2 diabetes mellitus, Hypertension

## Abstract

**Background:**

Hypertension and type 2 diabetes are associated with each other, and their coexistence is linked to diabetes-related complications such as stroke, coronary artery disease, kidney disease, retinopathy and diabetic foot. This study aimed to determine the prevalence, awareness and control of hypertension and factors associated with hypertension among people with type 2 diabetes mellitus (T2DM) in Bangladesh.

**Methods:**

A cross-sectional and retrospective study was conducted in 2017, and data from 1252 adults with T2DM were collected from six hospitals that specialise in diabetes care. These hospitals provide primary, secondary and tertiary healthcare and cover the rural and urban populations of Bangladesh. Cross-sectional data were collected from patients via face-to-face interviews, and retrospective data were collected from patients’ past medical records (medical passport), locally known as the patients’ guidebook or record book. The associations between hypertension and its related factors were examined using the bootstrapping method with multiple logistic regression to adjust for potential confounders.

**Results:**

The mean age of participants was 55.14 (± 12.51) years. Hypertension was found to be present among 67.2% of participants, and 95.8% were aware that they had it. Of these, 79.5% attained the blood pressure control. The mean duration of diabetes was 10.86 (± 7.73) years. The variables that were found to be related to hypertension include an age of above 60 years, physical inactivity, being overweight or obese, a longer duration of diabetes and chronic kidney disease.

**Conclusion:**

The prevalence of hypertension as well as its awareness and control were very high among people with known type 2 diabetes. As there is a strong relationship between hypertension and diabetes, patients with diabetes should have their blood pressure regularly monitored to prevent major diabetes-related complications.

## Background

Hypertension is a major non-communicable disease and is identified as a global disease burden that is ranked as the third-largest cause of disability-adjusted life years [1]. Globally, there were 972 million hypertensive adults in the year 2000, and this number is on track to increase by 60% to 1.56 billion by 2025 [1]. It has been reported that people with hypertension are twice as likely to develop cardiovascular disease, four times as likely to develop congestive heart failure and seven times as likely to develop cerebrovascular disease or stroke when compared to non-hypertensive subjects [2].

Diabetes mellitus and hypertension are inter-related diseases that strongly promote the spread of atherosclerotic cardiovascular disease within populations [3]. Hypertension prevalence is doubled in the presence of diabetes and is associated with 35 to 75% of diabetic cardiovascular and renal complications [4]. In addition, it also contributes to diabetic retinopathy, which is the primary cause of newly diagnosed blindness [3]. When a patient has diabetes, there is an accelerated formation of non-enzymatic advanced glycosylation products that accumulate in the vessel wall proteins, causing vascular rigidity and resistance, which ultimately leads to hypertension. Additionally, in mild-to-moderate hyperglycaemia, there is increased retention of sodium, which leads to an increase in the total exchangeable sodium and blood pressure [5]. Another plausible explanation is that the upregulation of the renin-angiotensin-aldosterone system in diabetes has a direct effect on hypertension [6]. Thus, diabetes and hypertension have an epidemiological and pathophysiological link, and knowledge of this link will not only help with the development of early treatment strategies but will also assist with prevention.

The population of Asian, especially South Asia, is facing an increasing burden from hypertension (ranging from 50% in India [7] to 75% in Pakistan [8]) among people with type 2 diabetes mellitus (T2DM). The burden of hypertension among patients with T2DM is also high in other regions (Korea (55%) [9], Nigeria (60%) [10] and Saudi Arabia (54.2–78.1%) [11, 12]). As of 2013, of the five South Asian countries with the highest prevalence of T2DM, Bangladesh Ranked second [13]. A report by the International Diabetes Federation stated that 7,926,300 cases of diabetes were recorded in Bangladesh [14]. A systematic review conducted in 2016 showed that the prevalence of T2DM in Bangladesh was 7.4% [15]. However, there has been a lack of studies in Bangladesh related to hypertension among people with T2DM. A study conducted in 1998 addressed hypertension among non-insulin-dependent subjects with diabetes mellitus and impaired glucose tolerance. This study reported that the proportion of people with systolic and diastolic hypertension was 32.2 and 13.6%, respectively [16]. According to the Bangladesh Demographic Health Survey 2011, the prevalence of T2DM in Bangladesh was 9.2% and among them 38.7% had hypertension [17]. Another study that was conducted in 2015, on the general population in Bangladesh, showed that 57.4% of patients with diabetes had hypertension compared to that of 15.6% of non-diabetic participants [18]. The above data shows an increasing trend in hypertension among T2DM patients in Bangladesh.

In recent years, along with the well-established pathophysiological link between T2DM and hypertension, the rapid epidemiological transition (rapid urbanisation, increased life expectancy, unhealthy diet and lifestyle changes) has led to an increase in hypertension among people with T2DM in Bangladesh. Thus, it is essential that patients and healthcare workers are aware of the co-existence of hypertension and T2DM to ensure that there is tight monitoring of blood pressure (BP) and blood sugar levels. To ensure good regulation, it is quintessential that an updated prevalence of hypertension, it’s awareness and control, and the factors associated with hypertension in patients with T2DM is available. Thus, this study aimed to determine the prevalence of hypertension and its associated factors among people with T2DM in Bangladesh.

## Methods

### Study design and sampling

A cross-sectional and retrospective study was conducted in 2017 in Bangladesh. Data were collected from outpatient department of six selected hospitals across the country that are under the umbrella of the Diabetic Association of Bangladesh (BADAS). All hospitals under BADAS are locally known as diabetes hospitals, as their primary focus is to treat patients with diabetes and its related complications. These hospitals are operated privately, but they provide healthcare services on a not-for-profit basis (i.e. their charged fee is lower than that of other private hospitals). These hospitals are located both outside and inside metropolitan areas across the country. The hospitals outside the metropolitan areas provide primary and secondary level healthcare services. However, within the metropolitan areas, the selected hospitals are the central hospitals that deliver primary, secondary and tertiary healthcare. The selection of the specific hospitals in this study made it possible to recruit a heterogeneous sample in terms of participants’ residential areas and socio-economic status.

BADAS has created a sustainable model of health care for people with diabetes and the general population. Most of the diabetic patients in Bangladesh prefer to visit hospitals under BADAS because of the availability of endocrinologists and diabetes-centred care and because the quality of facilities is higher than those in public hospitals. Thus, despite the fact that the participants in our study do not represent all people with T2DM in Bangladesh, they represent the majority of them. Hospitals under BADAS were purposively selected for data collection, as all patients are provided a record book or medical passport, which keeps patients’ records of laboratory test results, medications, complications and comorbidities.

It was calculated that a sample size of 1252 participants would be required to ensure accurate results. The primary outcome of this study was to determine haemoglobin A1c (HbA1c) levels and diabetes control [19], and the secondary outcome was to determine the prevalence of hypertension and its control among T2DM. Hence, to ensure that the accuracy in results was maintained for the secondary outcome of hypertension, the sample size was recalculated. This was done using a 5% significance level, 3.5% margin of error, 57.4% prevalence of hypertension among those with T2DM in Bangladesh and a design effect of 1.5 [20]. This provided a required sample size of 1150 participants. Thus, using data from 1252 participants ensured a greater level of accuracy than was required by the calculation.

During each day of data collection, around 10 patients were recruited from each hospital’s general outpatient department. The data collectors started the day by randomly selecting a patient from the first K patients attending the hospital and invited them to participate. The value of K was determined by dividing the total estimated hospital outdoor attendance each day by 10. After that, every Kth patient was approached. If this patient declined to participate in the study or did not have T2DM, the next patient was invited. The recruitment continued for 6 months, from Saturday to Thursday each week. The inclusion criteria were as follows: patients were over 18 years of age, were registered with BADAS and had had T2DM for over 1 year. The study excluded people suffering from other types of diabetes.

### Data collection

A structured questionnaire was developed based on published literature and different standardised questionnaires [21–23]. The questionnaire was pretested using a pilot study conducted in one of the selected tertiary hospitals. After getting informed written consent, the pretested questionnaire was used to interview each patient**.** A PhD research student and two data collectors were recruited and trained for data collection.

Data on sociodemographic characteristics, mental health (anxiety and depression), diabetes-related medical history and lifestyle behaviours were collected. The Global Physical Activity Questionnaire (GPAQ) was used to assess physical activity level [24]. The Patient Health Questionnaire (PHQ-2) [21] and Generalised Anxiety Disorder (GAD-2) [25] scales were used to evaluate depression and anxiety respectively. Participants were measured with their shoes off and in light clothes to determine their weight and height, and waist and hip measurements were also obtained. Either a medical officer or a registered nurse measured the blood pressure (BP) of each participant twice, at a 15-min interval, using mercury sphygmomanometer and stethoscope with participants sitting in a relaxed position and their left arm at rest on a table.

The following variables were extracted from medical passports or clinical records: most recent HbA1c level, lipid profiles, most recent estimated glomerular filtration rate (GFR), diagnosis of hypertension, medications used, and history of macro-vascular (coronary artery disease (CAD), stroke and diabetic foot) and micro-vascular (nephropathy, retinopathy and neuropathy) complications. For CAD and retinopathy, patients’ past medical records, past documented diagnoses, medications or past procedures were used to extract the data. Research Electronic Data Capture (REDCap) was used to collect and manage the data [26].

### Operational definition

Participants’ household income was categorised as follows: up to 20,000 Bangladeshi Taka (BDT), 21,000 to 60,000 BDT and over 60,000 BDT [19]. The monthly income was converted to USD using the mid-year currency conversion for 2017, which was 1 USD = 80 BDT [19]. At least 150 min of moderate to vigorous physical activity per week was considered as active [22]. Both PHQ-2 and GAD-2 had Likert scale questions with four options each [23, 25]. A patient with a total score of three or more was identified as having depression or anxiety. The body mass index (BMI) was categorised as follows: < 18.50 kg/m^2^ = underweight; 18.50 to 22.99 kg/m^2^ = normal; 23.00 to 27.49 kg/m^2^ = overweight and ≥ 27.5 = obese [27–29]. High waist–hip ratio was defined as > 0.90 for men and > 0.85 for woman [30]. Hypertension was defined by BP readings (either systolic ≥140 mmHg or diastolic ≥90 mmHg) or by using a documented diagnosis of hypertension for T2DM patients taking antihypertension medications [31]. Based on HbA1c level, diabetes control was categorised as a good control (HbA1c < 7.0%) or a poor control (HbA1c ≥ 7.0%). Renal impairment was determined using the most recent estimated GFR of below 60 ml/min/1.73m^2^ and/or a documented diagnosis [32].

### Ethical approval

This study has been approved by the Monash University Human Research Ethics Committee (ID: 1469), the Ethical Review Committee of the Bangladesh University of Health Sciences and the Ethical Review Committee of BADAS.

### Data management and analysis

Stata/SE version 15.0 was used for the data analysis. Data were summarised and presented as means (± standard deviation) for numerical data and relative percentages for categorical data. Chi-square tests and univariate logistic regression analysis were used to examine associations between hypertension and its associated factors. Potential variables were then identified using a clinical assessment, literature review and the variables with a *p*-value of less than 0.15 from the univariate analysis [19]. Of these, three variables had missing observations: BMI (8.6%, *n* = 108), waist–hip ratio (19.6%, *n* = 245) and glycaemic control (HbA1c 20.1%, *n* = 252).

Missing data were imputed five times using the chained equation method, creating five imputed samples [33, 34]. Then, the bootstrapping method [20, 35] with replacement was carried out to select the truly independent variables for hypertension. In the bootstrapping method, 5000 random samples of the size of the original sample (*n* = 1253) were drawn from each of the five imputed samples. This created 25,000 samples. A multiple logistic regression analysis was then performed for each of the 25,000 samples where all potential variables were added into the model, and the variables that appeared as significant were recorded. The percentage of the time that each variable was selected as significant (out of 25,000 samples) was calculated and variables were ranked from the highest to the lowest percentage. A multiple logistic regression model was then run using the variables that were selected in at least 40% of the bootstrap samples, which adjusted the effect of each variable with potential confounders.

## Results

**Fig. 1:**
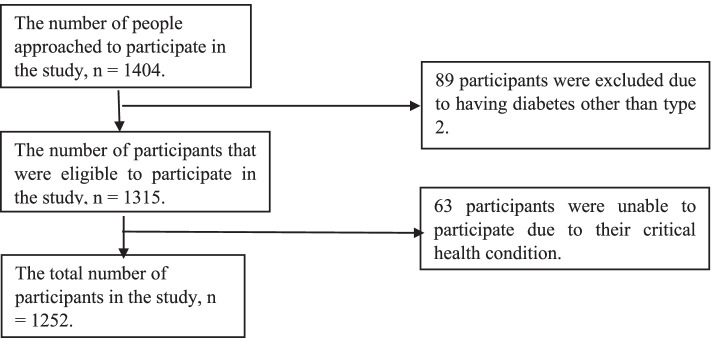
Flow-chart of selected participants

A total of 1404 people with diabetes were approached, but 89 of them had type 1 or gestational diabetes, and 63 of them declined to participate (Fig. 1). Among the 1252 participants, 64.91% were female. The mean age was 55.14 (± 12.51) years, while the mean duration of diabetes was 10.86 (± 7.73) years.

**Fig. 2:**
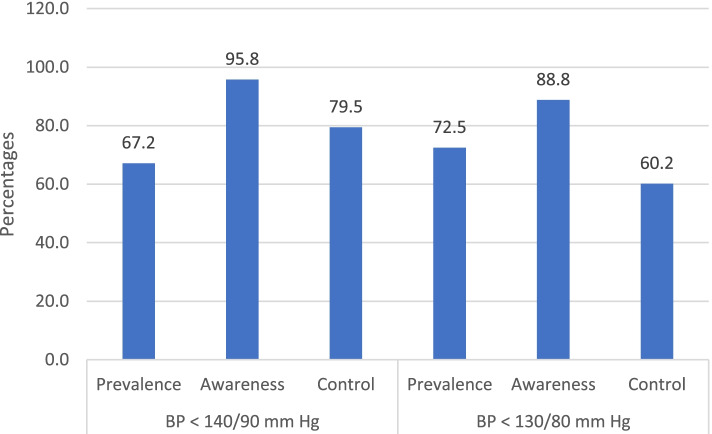
Hypertension awareness, prevalence and control among people with T2DM based on BP < 140/90 mmHg and < 130/80 mmHg. Note: Hypertension awareness: among all participants with hypertension; Hypertension control: among participants receiving hypertension treatment

**Fig. 3:**
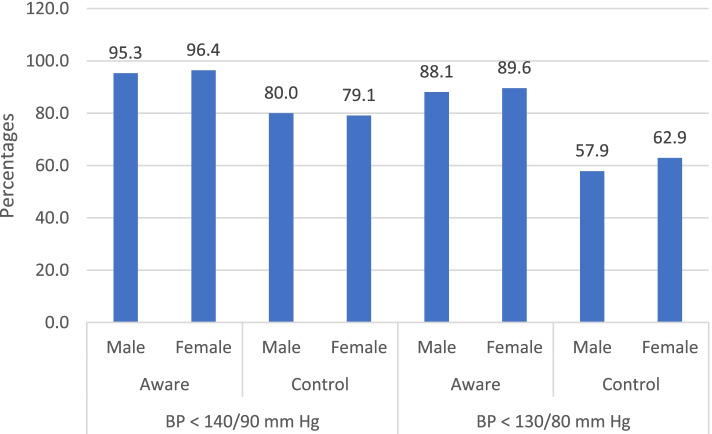
Hypertension awareness and control for male and female patients based on BP < 140/90 mmHg and < 130/80 mmHg. Note: Hypertension awareness: among all participants with hypertension; Hypertension control: among participants receiving hypertension treatment

Using BP cut point of 140/90 mmHg, as recommended by most guidelines of hypertension treatment, 67.2% (842/1252) of the participants in this study had hypertension. Of those with hypertension, 95.8% (807/842) were aware that they had hypertension (informed either by a doctor or nurse on previous occasions). The remaining 4.2% (*n* = 35/842) were unaware that they had it, but upon measuring their blood pressure during this present study it was found to be classified as being high. Furthermore, of those who were previously aware that they had hypertension, 79.5% (*n* = 642/807) had controlled hypertension, when measured for the present study. However, using a more aggressive BP cut point of 130/80 mmHg, as recommended for patients with diabetes, hypertension prevalence, awareness and control were 72.5% (*n* = 909/1252), 88.8% (807/909), and 60.2% (*n* = 486/807) respectively (Fig. 2). There was no significant difference of hypertension control between male and female (for BP cut point of 140/90 mmHg: male 80% vs female 79.1%, *p*-value = 0.737; and for BP cut point of 130/80 mmHg: male 57.9% vs female 62.9%, *p*-value = 0.151). The prevalence of hypertension awareness for male and female was 95.3 and 96.4% (*p*-value = 0.435) respectively for BP cut point of 140/90 mmHg, and that was 88.1 and 89.6% (*p*-value = 0.495) for BP cut point of 130/80 mmHg respectively (Fig. 3).

The prevalence of hypertension was higher in those over 60 years (78%) as compared to patients 60 years and below (57.8%) (*p*-value < 0.001). Hypertension prevalence was similar between male and female (male: 63.1% vs female: 64.9%, *p*-value = 0.308). The prevalence of hypertension was also significantly higher among people who resided in urban areas, were physically inactive, were obese or had a high waist–hip ratio (*p*-value < 0.001). The presence of hypertension was also significantly higher among people who never smoked (66.1%) compared to those who smoke presently (48.9%) and did in the past (63.9%), with a *p*-value of 0.004 (Table 1).

**Table 1:** Demographic, socioeconomic, anthropometric, and clinical baseline characteristics according to hypertension

Variables	Hypertension		
	No % (n) 35.6% (446)	Yes % (n) 64.4% (807)	*P*-value
Age:			
≤ 60 years	42.1 (356)	57.9 (489)	< 0.001
≥ 61 years	22.1 (90)	78.0 (318)	
Gender:			
Female	34.1 (195)	64.9 (377)	0.308
Male	36.9 (251)	63.1 (430)	
Area of residency:			
Rural	45.8 (154)	54.2 (182)	< 0.001
Urban	31.8 (292)	68.2 (625)	
Education:			
Illiterate/primary	31.7 (127)	68.2 (273)	0.117
Secondary	37.6 (207)	63.4 (359)	
Graduate	39.0 (112)	61.0 (175)	
Household income:			
≥ 61,000 BDT	31.4 (97)	68.6 (212)	0.167
21,000–60,000 BDT	36.0 (179)	64.0 (318)	
≤ 20,000 BDT	38.0 (170)	62.0 (277)	
Physical activity:			
Active	41.0 (251)	59.0 (361)	< 0.001
Inactive	30.4 (195)	69.6 (446)	
Smoking status:			
Never smoked	33.9 (315)	66.1 (614)	
Smoked in the past	36.1 (83)	63.9 (147)	0.004
Current smoker	51.1 (48)	48.9 (46)	
Body mass index:			
Normal	46.5 (135)	53.4 (155)	< 0.001
Overweight	38.5 (209)	61.5 (326)	
Obese	24.9 (81)	75.1 (244)	
Waist/hip ratio:			
Normal	52.7 (88)	47.1 (49)	< 0.001
High	36.7 (331)	63.3 (572)	
Duration of diabetes:			
≤ 10 years	42.4 (303)	57.6 (412)	< 0.001
≥ 11 years	26.6 (143)	73.4 (395)	
Mode of treatment:			
OHA	40.9 (174)	59.7 (258)	0.012
Combination	33.1 (272)	69.9 (507)	
Glycaemic control (HbAlc):			
< 7%	39.6 (22)	60.4 (110)	0.783
≥ 7%	38.5 (315)	61.5 (504)	
Chronic kidney disease:			
No	44.5 (367)	55.5 (457)	< 0.001
Yes	18.4 (79)	81.6 (350)	
Depression:			
No	40.1 (318)	59.5 (467)	< 0.001
Yes	27.3 (128)	72.6 (340)	
Anxiety:			
No	35.8 (402)	64.2 (721)	< 0.001
Yes	33.8 (44)	66.1 (86)	
Macrovascular-Microvascular complications:			
No complications	55.5 (254)	44.5 (204)	< 0.001
Microvascular complications	29.7 (77)	70.3 (182)	
Macrovascular complications	33.9 (63)	66.1 (123)	
Both complications	14.9 (52)	85.1 (298)	

BDT: Bangladeshi Taka; Body mass index: < 18.50 kg/m^2^ = underweight; 18.50 to 22.99 kg/m^2^ = normal; ≥ 23.00 to 27.49 kg/m^2^ = overweight and ≥ 27.5 obese. High waist-hip ratio > 0.90 for men and > 0.80 for women. Adequate physical activity (Global Physical Activity Questionnaire): > 150 min of moderate to vigorous activity per week. OHA: Oral hypoglycaemic agent; macro-vascular complications: Coronary artery disease (CAD), stroke and diabetic foot; microvascular complications: Nephropathy, retinopathy and neuropathy; chronic kidney disease: estimated glomerular filtration rate < 60 ml/min/1.73m^2^ Depression/anxiety: The PHQ-2 and GAD-2 scales score ≥ 3

According to the findings, people who have had diabetes for over 10 years, used insulin either solely or in combination with an oral hypoglycaemic agent (OHA) or have a history of chronic kidney diseases showed an increased prevalence of hypertension (*p* < 0.001). Also, the presence of hypertension was higher among people who had depression, anxiety or micro- and macro-vascular complications (*p* < 0.001) (Table 1).

**Fig. 4:**
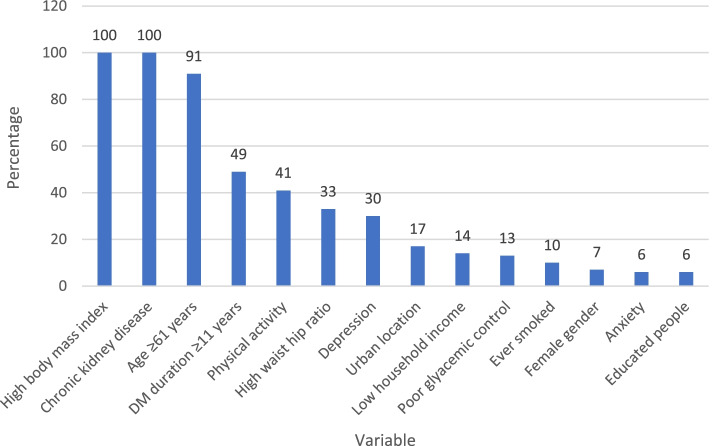
The percentage of the time that variables were significant for hypertension in bootstrap analysis

Bootstrap method was used to find the potential variables for multiple logistic regression analysis. Being overweight or obese along with having chronic kidney disease and being older than 60 years appeared as significant in over 90% of bootstrap samples. However, a diabetes duration of over 10 years and physical inactivity appeared as significant in 41 and 49% of samples, respectively. While waist–hip ratio and depression appeared in 33 and 30% of the samples. The remaining variables appeared as significant in less than 30% of the samples. The variables that appeared in at least 40% of the bootstrap samples were added to the multiple logistic regression to adjust for potential confounders (Fig. 4).

The results from multiple logistic regression analyses showed that being above 60 years (OR: 1.7, 95% CI: 1.3, 2.4), overweight (OR: 1.8, 95% CI: 1.3, 2.5), obese (OR: 3.2, 95% CI: 2.26, 4.8) or physically inactive (OR: 1.3, 95% CI: 1.0, 1.7) increased the odds of hypertension. Furthermore, higher odds were observed among patients with chronic kidney disease (OR: 3.1, 95% CI: 2.2, 4.3) and who had diabetes for over 10 years (OR: 1.4, 95% CI: 1.0, 1.8) (Table 2).

**Table 2:** Simple and multiple logistic regression association of hypertension with demographic, lifestyle and clinical characteristics

Variables	Hypertension					
	Simple logistic regression			Multiple logistic regression		
	Odds ratio	95%CI	*P*-value	Odds ratio	95% CI	*P*-value
Age (ref: ≤60 years):						
≥ 61 years	2.6	2.0, 3.4	< 0.001	1.74	1.3, 2.4	< 0.001
Gender (ref: male):						
Female	1.1	0.9, 1.5	0.30	1.00	0.8, 1.3	0.980
Area of residency (ref: rural):						
Urban	1.8	1.4, 2.3	< 0.001	–	–	–
Education (ref: illiterate/primary):						
Secondary	0.8	0.6, 1.1	0.121	–	–	–
Graduate	0.8	0.5, 0.9	0.049	–	–	–
Household Incomes (ref: ≥61,000 BDT):						
21,000–60,000 BDT	0.1	0.6, 1.1	0.179	–	–	–
≤ 20,000 BDT	0.7	0.5, 1.0	0.061	–	–	–
Physical activity (ref: active):						
Inactive	1.5	1.4, 2.3	< 0.001	1.3	1.0,1.7	0.030
Smoking (ref: never smoked):						
Smoked in the past	0.9	0.6, 1.2	0.533	–	–	–
Current smoker	0.5	0.3, 0.8	0.001	–	–	–
Body Mass Index (ref: normal):						
Overweight	1.4	1.0, 1.9	0.025	1.8	1.3, 2.5	< 0.001
Obese	2.6	1.9, 3.7	< 0.001	3.3	2.3, 4.8	< 0.001
Waist/hip (ref: normal):				–	–	–
High	1.9	1.3, 2.9	< 0.001			
DM duration (ref: ≤ 10 years):						
≥ 11 years	2.0	1.6, 2.6	< 0.001	1.4	1.0, 1.8	0.024
Mode of treatment (ref: OHA):						
Insulin	0.6	0.4, 1.0	0.050	–	–	–
Combination	1.5	1.2, 1.9	< 0.001	–	–	–
Glycaemic control (HbAlc) (ref < 7%):						
≥ 7%	1.0	0.8, 1.5	0.783	–	–	–
Chronic kidney disease (ref: normal):						
Chronic kidney disease	3.6	2.7, 4.7	< 0.001	3.1	2.2, 4.3	< 0.001
Depression (ref: no depression):						
Depression	1.8	1.4, 2.3	< 0.001	–	–	–
Anxiety (ref: no anxiety):						
Anxiety	1.1	0.7, 1.5	0.066	–	–	–
Macrovascular-Microvascular complications (ref: normal):						
Microvascular complications	2.9	2.1, 4.1	< 0.001	–	–	–
Macrovascular complications	9.4	1.7, 3.5	< 0.001	–	–	–
Both complications	7.1	5.0, 10.1	< 0.001	–	–	–

BDT: Bangladeshi Taka; DM: Diabetes mellitus, OHA: Oral hypoglycaemic agent; the body mass index: < 18.50 kg/m^2^ = underweight; 18.50 to 22.99 kg/m^2^ = normal; ≥ 23.00 to 27.49 kg/m^2^ = overweight and ≥ 27.5 obese. High waist-hip ratio: > 0.90 for men and > 0.80 for women**;** adequate physical activity (Global Physical Activity Questionnaire): > 150 min of moderate to vigorous activity per week; macro-vascular complications: coronary artery disease (CAD), stroke and diabetic foot; microvascular complications: nephropathy, retinopathy and neuropathy; chronic kidney disease: estimated glomerular filtration rate < 60 ml/min/1.73m^2^ and depression/anxiety: the PHQ-2 and GAD-2 scales score ≥ 3

## Discussion

This study was conducted to explore hypertension prevalence, awareness and control, and factors related to hypertension among T2DM patients in diabetes hospitals in Bangladesh. The study demonstrated that 67.2% of the participants had hypertension. The prevalence of hypertension among patients with diabetes was higher in the current study than previously reported in Bangladesh (57.4%) [36] or other South Asian countries (Indian 50% [7], Bhutan 54% [37] and Nepal 36.7% [38]) except for Pakistan (75%) [8].

The prevalence of hypertension awareness among hypertensive T2DM in this study was 95.8%, and among them 79.5% had it controlled. The data on hypertension awareness and its control among T2DM patients is scarce in the South Asian countries. However, a multi-country study conducted from 2003 to 2009 on the general population in India, Pakistan and Bangladesh showed that 40.4% of the hypertensive patients were aware of it and 12.9% had it controlled [39]. In comparison with other regions, the hypertension awareness and control among T2DM in this current study was higher than that reported in China (57.5%) [40] and in the USA (53%) [41] and Malaysia (33.3%) [42] respectively. The high awareness and control of hypertension in this study might be because of the policy of the diabetes hospitals that requires blood pressure to be measured during each diabetes check-up visit and the regular onsite and camp diagnosis undertaken under BADAS to create awareness of hypertension among patients with known diabetes. Also, the free investigation and treatment the diabetes hospitals provide for the financially disadvantaged patients, making it more accessible and affordable [43]. The prevalence of hypertension awareness and control among participants with known T2DM in this study were in good levels as compared to global standards. However, a further hypertension control among hypertensive T2DM may be achieved by strengthening counselling, educating and empowering in self-monitoring. In addition, hypertension control among hypertensive T2DM may be required a more aggressive goal of BP < 130/80 mmHg as recommend for patients with diabetes mellitus.

The multivariable analysis in the current study showed that older age, physical inactivity, being overweight or obese, longer duration of diabetes and chronic kidney disease are related to hypertension among people with T2DM. As previous evidence suggests, our study demonstrated that being physically inactive was associated with an increased prevalence of hypertension [37, 44] and insufficient physical activity was highly prevalent among T2DM in Bangladesh [45]. The findings of our study therefore amplify the need to direct efforts and resources to increase the physical activity levels of people with T2DM in Bangladesh. This study’s results further show that being overweight or obese might increase the odds of hypertension, which is supported by previous studies [46, 47]. In recent decades, obesity among adults has been on the rise in Bangladesh and has reached a point where one in five adults is now obese [48]. Therefore, maintaining a healthy weight is essential for people with diabetes in the country. As for the duration of diabetes, this current study confirmed the previous finding that having diabetes for a long time is significantly related to hypertension [49]. The presence of chronic kidney disease was also significantly associated with hypertension in this study. Many scientific studies have clarified this relationship [50] and have established their pathophysiological link [51, 52]. However, it is important to note that hypertension is a well-known risk factor for kidney disease, as well as the other way around.

This study has strengths and limitations. First, it used a relatively large sample that came from six diabetes hospitals that provide primary, secondary and tertiary healthcare and cover rural and urban populations. Second, in addition to a large sample size, this study used validated questionnaires, which provides a valuable repository of data that merits dissemination. Third, the investigation of various demographic, behavioural and clinical factors added strength to the study. A limitation of this study was that it was cross-sectional and retrospective in its design, which only allows for the implication of association, rather than causation. The unavailability of data on the treatment of hypertension among hypertensive T2DM patients was another limitation. Finally, a major limitation is that the findings of this study are not generalizable to diabetes patients not receiving care at BADAS hospitals.

## Conclusion

Considering the high proportion of hypertension among patients with diabetes and the close pathophysiological association between T2DM and hypertension, patients with T2DM should have regular monitoring of blood pressure to diagnose and control hypertension. The factors related to hypertension among patients with T2DM are the same as those found in other studies, and this study highlights the need for lifestyle interventions to decrease obesity levels and increase physical activity. The BADAS hospitals’ policy of mandatory blood pressure monitoring in each diabetes check-up visit and a full or partial fee waiver for investigation and treatment for financially disadvantaged patients may be a key factor for high levels of hypertension awareness and controls among T2DM patients. This study suggests and highlights the successful policy implementation of hypertension management at hospitals in Bangladesh and other similarly positioned countries in accordance with international guidelines [53].

## Data Availability

The data sets generated during and/or analysed during the current study are available from the corresponding author upon reasonable request. Prior permission from the respective hospitals was obtained for collecting data such as lipid profiles, HbA1c level, and diabetes related macro- and microvascular complications from hospital records retrospectively.

## Abbreviations

T2DM: Type 2 diabetes mellitus
BP: Blood pressure
BADAS: Diabetic Association of Bangladesh
GPAQ: Global Physical Activity Questionnaire
PHQ-2: Patient Health Questionnaire
GAD-2: Generalised Anxiety Disorder
HbA1c: Haemoglobin A1c
GFR: Glomerular filtration rate
CAD: Coronary artery disease
REDCap: Research Electronic Data Capture
BDT: Bangladeshi Taka
USD: Unites States Dollar
BMI: Body mass index
OHA: Oral hypoglycaemic agent
OR: Odds ratio
